# Distribution of siderophore gene systems on a *Vibrionaceae* phylogeny: Database searches, phylogenetic analyses and evolutionary perspectives

**DOI:** 10.1371/journal.pone.0191860

**Published:** 2018-02-14

**Authors:** Sunniva Katharina Thode, Ewelina Rojek, Mikolaj Kozlowski, Rafi Ahmad, Peik Haugen

**Affiliations:** 1 Department of Chemistry and Center for Bioinformatics (SfB), Faculty of Science and Technology, UiT − The Arctic University of Norway, Tromsø, Norway; 2 Department of Natural Sciences and Technology, Faculty of Education and Natural Sciences, Inland Norway University of Applied Sciences, Hamar, Norway; Academia Sinica, TAIWAN

## Abstract

Siderophores are small molecules synthesized and secreted by bacteria and fungi to scavenge iron. Extracellular ferri-siderohores are recognized by cognate receptors on the cell surface for transport over membranes. Several siderophore systems from *Vibrionaceae* representatives are known and well understood, e.g., the molecular structure of the siderophore, the biosynthesis gene cluster and pathway, and the gene expression pattern. Less is known about how these systems are distributed among the ~140 *Vibrionaceae* species, and which evolutionary processes contributed to the present-day distribution. In this work, we compiled existing knowledge on siderophore biosynthesis systems and siderophore receptors from *Vibrionaceae* and used phylogenetic analyses to investigate their organization, distribution, origin and evolution. Through literature searches, we identified nine different siderophore biosynthesis systems and thirteen siderophore receptors in *Vibrionaceae*. Homologs were identified by BLAST searches, and the results were mapped onto a *Vibrionaceae* phylogeny. We identified 81 biosynthetic systems distributed in 45 *Vibrionaceae* species and 16 unclassified *Vibrionaceae* strains, and 409 receptors in 89 *Vibrionaceae* species and 49 unclassified *Vibrionaceae* strains. The majority of taxa are associated with at least one type of siderophore biosynthesis system, some (e.g., aerobactin and vibrioferrin) of which are widely distributed in the family, whereas others (i.e., bisucaberin and vibriobactin) are found in one lineage. Cognate receptors are found more widespread. Phylogenetic analysis of three siderophore systems (piscibactin, vibrioferrin and aerobactin) show that their present-day distribution can be explained by an old insertion into *Vibrionaceae*, followed mainly by stable vertical evolution and extensive loss, and some cases of horizontal gene transfers. The present work provides an up to date overview of the distribution of siderophore-based iron acquisition systems in *Vibrionaceae*, and presents phylogenetic analysis of these systems. Our results suggest that the present-day distribution is a result of several evolutionary processes, such as old and new gene acquisitions, gene loss, and both vertical and horizontal gene transfers.

## Introduction

Siderophores represent a group of relatively small, and low molecular weight secondary metabolites with high-affinity binding potential to ferric iron [1]. They are produced and secreted by a broad range of microorganisms (e.g., bacteria and fungi), and some plants. Under low iron conditions, such as in aquatic environments or inside a vertebrate host, e.g., bacteria must use highly specific strategies to acquire iron and other essential micronutrients [2,3]. To overcome iron starvation, siderophores are synthesized and secreted to their surroundings where they chelate ferric iron. Once bound, the ferric iron-siderophore complexes are recognized by siderophore receptors, and transported over the membrane by ABC transporters using TonB complexes as energy transducers.

Interestingly, bacteria produce siderophores of several major classes, each of which can have a diverse set of molecular structures, presumably because production of unique siderophores can provide individual bacteria with an advantage in the competition with others [4]. For example, polymicrobial studies have shown that siderophores from one species can inhibit growth or functions of other species, e.g. low concentrations of avaroferrin from *Shewanella algae* inhibit swarming of *Vibrio alginolyticus* and a siderophore from *Pseudomonas fluorescens* inhibits growth of *Vibrio anguillarum* [5,6]. Such kin discrimination strategy can however be bypassed by “cheaters”, i.e., bacteria expressing receptors on their surface with affinity to siderophores produced by others [7]. This mechanism is also known as exogenous or xeno-siderophore utilization. So surely, there must be a constant battle between microorganisms for available iron, and they can produce (i) own siderophores and the respective receptors, and/or (ii) “cheating” receptors for utilization of siderophores produced by others.

We have in this work, studied siderophore biosynthesis systems and their respective receptors from the *Vibrionaceae* family. *Vibrionaceae* represents a large and diverse group of Gram-negative Gammaproteobacteria, and the evolutionary relationships between many of the approximately 140 different species were recently updated by Sawabe and coworkers [8]. Representatives of this family have been heavily studied, typically due to their ability to cause serious diseases in humans or animals.

The causative agent of the human disease cholera, *Vibrio cholerae*, is the most famous *Vibrionaceae* representative. *V*. *cholerae* produces the catechol siderophore vibriobactin using proteins encoded by *vibABCDEFH* [9,10]. Ferric iron-vibriobactin complexes are recognized by the receptor ViuA [11]. Moreover, *V*. *cholerae* can “cheat” on derivatives of enterobactin (produced by e.g., *Escherichia coli*) using the receptors IrgA and VctA [12], fluvibactin (synthesized by *Vibrio fluvialis*) using the ViuA, VctA and IrgA receptors, and finally ferrichrome by using the FhuA receptor [12–14]. *Vibrio vulnificus* represents another significant human pathogen [15]. This bacterium produces the catechol siderophores vulnibactin by using proteins encoded by the gene cluster VV2_0830—VV2_0844 [16], and recognizes ferri-vulnibactin via the VuuA receptor [17]. It has also been proposed that *V*. *vulnificus* produces an uncharacterized hydroxamate siderophore, and an uncharacterized catechol siderophore using, in part, same genes as for vulnibactin [16,18]. Finally, *V*. *vulnificus* can transport and utilize aerobactin (IutA receptor) [19], deferoxamine B (DesA receptor) [20,21] and vibriobactin [22]. The human pathogen *Vibrio parahaemolyticus* [23] produces the carboxylate siderophore named vibrioferrin (encoded by *pvsABDE*) [24]. Vibrioferrin is sensitive to photolysis and has a lower affinity for iron compared to other catechol-type siderophores in vibrios. Ferri-vibrioferrin is recognized and transported over the membranes using the receptor PvuA [25]. *V*. *parahaemolyticus* can “cheat” using the exogenous siderophores enterobactin, aerobactin, ferrichrome and possibly vibriobactin and fluvibactin [22,26–29]. *V*. *alginolyticus* is an emerging foodborne pathogen that causes gastroenteritis and peritonitis in humans [30]. The B522 strain contains the vibrioferrin biosynthesis cluster [5,31], and can also utilize siderophores synthesized by *V*. *cholerae*, *V*. *fluvialis* and *V*. *parahaemolyticus* and ferrichrome [32,33].

Several *Vibrionaceae* fish pathogens have been studied with respect to siderophore production and utilization, e.g., *V*. *anguillarum*, a pathogen causing haemorrhagic septicemia in fish, bivalves and crustaceans [34], *Aliivibrio salmonicida*, causing cold-water vibriosis in Atlantic salmon at low seawater temperatures [35,36], *Photobacterium damselae subsp*. *piscicida* [37,38], and *V*. *alginolyticus* [30]. Depending on strain, *V*. *anguillarum* can synthesize and utilize the mixed catechol/hydroxamate siderophore anguibactin (only serotype O1 strain; biosynthesis encoded by *angABCE*^*B*^/_*G*_*MTHRNUD* and recognized by FatA receptor) [39,40], or vanchrobactin (found in all serotype O2 strains, some plasmid less O1 strains, and several other serotypes). Biosynthesis of the latter is encoded by *dapH* and *vabABCEFH* [41], and recognized by a receptor encoded by *fvtA* [42]). Anguibactin biosynthesis genes are located both on a conjugative plasmid named pJM1, and on chromosomes (*angABC* and *angE*) [40]. Intriguingly, for *V*. *anguillarum* strain 775 the presence of pJM1 and anguibactin coincides with the lack of vanchrobactin [43]. Its chromosome contains entire vanchrobactin gene cluster, except that *vabF* is interrupted by an RS1 transposon originating from pJM1. Closely related strains that lacks this plasmid produce vanchrobactin. The authors therefore hypothesize that vanchrobactin was produced by the bacterium prior to the acquisition of pJM1 (and thus the anguibactin cluster), and that production of vanchrobactin at some point was suppressed by inactivation of *vabF* since anguibactin has a higher affinity for iron. Moreover, *V*. *anguillarum* utilizes exogenous siderophores like enterobactin, ferrichrome and citrate [44,45]. *A*. *salmonicida* synthesizes and utilizes the di-hydroxamate siderophore bisucaberin (biosynthesis encoded by *bibABC* and recognized by the BitA receptor) [46,47]. It has been postulated that the siderophore production is vital for the virulence of *A*. *salmonicida*. This assumption is based on that production of significant amounts of bisucaberin is restricted to low temperature conditions (i.e., the bacterium only causes disease at low temperatures) [46]. Also, we recently showed that the genes responsible for bisucaberin production are highly up-regulated under low iron conditions and that the production is strongly regulated by Fur [48]. A system for aerobactin synthesis is in contrast not expressed, probably because the cluster is non-functional due to frameshift mutations and loss of the promotor [49]. The genome of *A*. *salmonicida* also encodes the deferroxamine B receptor DesA and the aerobactin receptor IutA [49]. The fish pathogen *P*. *damselae subsp*. *piscicida* produces the mixed carboxylate/hydroxamate siderophore piscibactin (encoded by *dapH* and *irp123459*), which is probably transported by FrpA [37,38,50]. The shrimp pathogen *Vibrio campbelli* produces the catechol siderophore amphi-enterobactin (biosynthesis encoded by *aebABCEG*), however the receptor has not been identified [51]. In addition, *Vibrionaceae* representatives may produce other siderophores such as amphibactins, deferroaxamines, trivanchrobactins, ochrobactins and probably several more. However, although the biosynthetic gene clusters responsible for production of these molecules are well known from other bacteria, they may not have been conclusively identified in *Vibrionaceae*. In *Vibrio campbellii* DS40M4, the same gene cluster is responsible for production of both vanchrobactin and trivanchrobactin, but the main determinant that regulates which of them is produced remains unknown [52]. Payne and co-workers recently reviewed siderophore biosynthesis and utilization in *Vibrionaceae*, with a focus on vibrios [7]. This inspired us to use the existing knowledge to investigate the distribution and evolution of the different siderophore systems further. In this work, we first performed literature searches on *Vibrionaceae* siderophore gene systems, then we used this knowledge to search the databases for siderophore systems in all available *Vibrionaceae* genomes, and mapped the result onto a *Vibrionaceae* phylogenetic network. The evolution of individual siderophore biosynthesis systems and receptors was next studied by constructing phylogenetic trees based on amino acids datasets, and by comparing the resulting tree topologies to host trees. Through the presented work, we wish to broaden the perspective and existing knowledge on siderophore synthesis and utilization within the *Vibrionaceae* family.

## Materials and methods

### Data retrieval

Siderophore biosynthesis gene clusters and associated siderophore receptor genes in *Vibrionaceae* were identified by literature searches, and the corresponding protein sequences were retrieved from NCBI’s protein sequence databank. The literature search was done over several months during fall 2016. Updated RefSeq accession numbers for identified proteins with the ‘WP’ prefix (the ‘WP’ accession prefix was introduced to decrease redundancy in RefSeq, and has replaced the ‘YP’, ‘NP’ and ‘ZP’ prefixes) are presented in Tables 1 and 2. These sequences were next used as queries in BLASTp searches to find homologous protein sequences. BLASTp was run using the non-redundant protein database while restricting the search to the *Vibrionaceae* family (NCBI taxid: 641). The following criteria were used to decide if a siderophore biosynthesis gene cluster is present in any given species: (i) threshold values from BLASTp were set to ≥80% coverage and ≥50% identity, (ii) all proteins associated with a siderophore gene cluster must be present in the same species, (iii) pseudogenes were rejected, and (iv) BLASTp hits labelled “low quality protein” in the databases were excluded. Within-species variations were not considered because it would require extensive manual curation of a huge number of blast hits and database entries, which was not feasible to do as part of this study. Also, some of the siderophore pathways may share parts of the biosynthesis steps e.g., in the proposed pathways of anguibactin, vanchrobaction, vibriobactin and enterobactin synthesis, all involve synthesis of DHBA, later the four pathways split into unique steps. Siderophore synthesis pathways may therefore use common enzymes, or they may encode redundant enzymes. Such overlapping and redundancies of pathways were not specifically considered in this work.

**Table 1:** RefSeq accession numbers of known *Vibrionaceae* siderophore biosynthetic proteins.

Siderophore	Organism	Siderophore biosynthesis protein accession numbers	Ref
Aerobactin	*V*. *mimicus*	IucA(WP_000554936.1) IucB(WP_000033134.1) IucC(WP_000372426.1) IucD(WP_000401386.1)	[53]
Bisucaberin	*A*. *salmonicida*	BibA(WP_012549025.1) BibB(WP_012549026.1) BibC(WP_012549027.1)	[47]
Vibrioferrin	*V*. *parahaemolyticus*	PvsA(WP_015313675.1) PvsB(WP_015313676.1) PvsC(WP_015313677.1) PvsD(WP_015313678.1) PvsE(WP_015313679.1)	[24]
Vibriobactin	*V*. *cholerae*	VibA (WP_000654285.1) VibB (WP_000997093.1) VibC(WP_000245175.1) VibD(WP_000874996.1) VibE (WP_000205544.1) VibF (WP_000523394.1) VibH(WP_001880577.1)	[9,10]
Vanchrobactin	*Vibrio anguillarum*	DapH(WP_011154675.1) VabA(WP_064624836.1) VabB(WP_064624831.1) VabC(WP_043004165.1) VabE(WP_019281788.1) VabF (WP_019281791.1) VabH (WP_019281793.1)	[41]
Piscibactin	*P*. *damselae subsp*. *piscicida*	DapH (AKQ52526.1) Irp1(AKQ52532.1) Irp2(AKQ52531.1) Irp3(AKQ52533.1) Irp4(AKQ52534.1) Irp5(AKQ52536.1)	[37]
Anguibactin	*V*. *anguillarum*	AngA(WP_013857267.1) AngB(WP_013857270.1) AngC(WP_043004165.1) AngE(WP_013857269.1) AngB/G(WP_011154672.1) AngM(WP_011154633.1) AngT(WP_011154640.1) AngH(WP_011154645.1) AngR(WP_011154639.1) AngN(WP_011154642.1) AngU(WP_011154641.1) AngD(WP_011154670.1)	[40]
Vulnibactin	*V*. *vulnificus*	VV2_0830(WP_011081748.1) VV2_0831(AAO07755.1) VV2_0834(WP_011081751.1) VV2_0835(WP_011081752.1) VV2_0836(WP_011081753.1) VV2_0838/VenB(WP_011081755.1) VV2_0839(WP_011081756.1) VV2_0840(WP_011081757.1) VV2_0844(AAO07767.2)	[16]
Amphi-enterobactin	*V*. *campbellii*	AebG (WP_012127281.1) AebA(WP_041853223.1) AebC(WP_012127292.1) AebE(WP_012127293.1) AebB(WP_012127294.1) AebF(WP_041853220.1)	[51]

**Table 2:** RefSeq accession numbers of known *Vibrionaceae* siderophore receptor proteins.

Organism	Receptor	Transport	Ref
*V*. *mimicus*	IutA (WP_000843157.1)	Aerobactin	[53]
*A*. *salmonicida*	BitA (WP_012549028.1)	Bisucaberin	[47]
*V*. *parahaemolyticus*	PvuA (WP_057620147.1)	Vibrioferrin	[25]
*V*. *parahaemolyticus*	PeuA (WP_005479624.1)	Enterobactin	[27]
*V*. *cholerae*	ViuA (WP_000279435.1)	Vibriobactin Fluvibactin	[11]
*V*. *anguillarum*	FvtA (WP_019281795.1)	Vanchrobactin	[42]
*V*. *anguillarum*	FatA (WP_011154638.1)	Anguibactin	[54]
*V*. *cholerae*	VctA (WP_000350325.1)	Enterobactin Fluvibactin	[13]
*V*. *cholerae*	IrgA (WP_000086048.1)	Enterobactin Fluvibactin	[13]
*V*. *vulnificus*	VvuA (WP_015728225.1)	Vulnibactin	[17]
*V*. *cholerae*	FhuA (WP_053043596.1)	Ferrichrome	[14]
*V*. *furnissii*	DesA (WP_004725209.1)	Deferoxamine B	[55]
*P*. *damselae subsp*. *Piscicida*	FrpA (AKQ52529.1)	Piscibactin	[37]

### Mapping of siderophore systems onto a *Vibrionaceae* phylogenetic network

A *Vibrionaceae* host phylogeny was inferred based on sequence alignments of the genes *ftsZ*, *gap*, *gyrB*, *mreB*, *pyrH*, *recA*, *rpoA* and *topA*, provided by Dr. Sawabe [8]. SplitsTree4 [56] was used to concatenate the sequences to construct a multi locus sequence alignment (MLSA), and to generate an unrooted phylogenetic network. Settings were set to ‘NeighbourNet’ method with ‘uncorrected P’ distance. Presence/absence of siderophore biosynthesis and receptor genes were mapped onto the phylogenetic network. Only complete siderophore biosynthesis clusters are shown. The siderophore receptors were considered separately, and mapped onto the same network. Species with positive hits, but not included in the MLSA dataset, were placed onto the network based on the literature. “Unclassified” *Vibrionaceae* strains are not shown on the network, but can be found in S1 and S2 Tables.

### Phylogenetic analysis of siderophore biosynthesis systems and receptors

Amino acid sequences of proteins involved in siderophore biosynthesis were aligned using ClustalW [57]. Proteins belonging to same clusters were concatenated using SplitsTree4 [56] and exported to Fasta format, thus generating the final datasets. Next, Mega6 [58] was used to generate Maximum Likelihood (ML) trees based on the individual siderophore biosynthesis datasets. The robustness of nodes in the resulting phylogenies was tested by running Bootstrap analyses, using the ML method (2000 replicates, JTT substitution model, uniform rates, and ‘Complete deletion’ in gap handling).

To address inheritance of the siderophore biosynthesis systems, we next constructed host phylogenies of same taxa as those containing the siderophore systems. Host trees were based on concatenated datasets of the same eight genes as described above. ML-trees were constructed using the Tamura-Nei model [59], and all gaps and missing data were removed. Phylogenies of the vibrioferrin (PvsABCDE), piscibactin (Irp123459), and aerobactin (IucABCD) biosynthesis systems, and their corresponding MLSA host trees, were rooted on *Aliivibrio wodanis*, *Photobacterium profundum*, and *Grimontia hollisae*, respectively. The phylogeny of siderophore receptors was constructed essentially as described above. Briefly, amino acid sequences of homologous receptor sequences were aligned using ClustalW, and Mega6 [58] was then used to make ML-trees. Bootstrap analysis was done using the ML method, 2000 pseudoreplicates, the JTT model, uniform rates, and complete deletion of gaps. Corresponding MLSA trees were constructed as described for the cluster. The receptor phylogenies were compared to host trees, which were constructed as described above.

## Results and discussion

### Compilation of siderophore biosynthesis gene cluster sequences from *Vibrionaceae*

**Fig 1:**
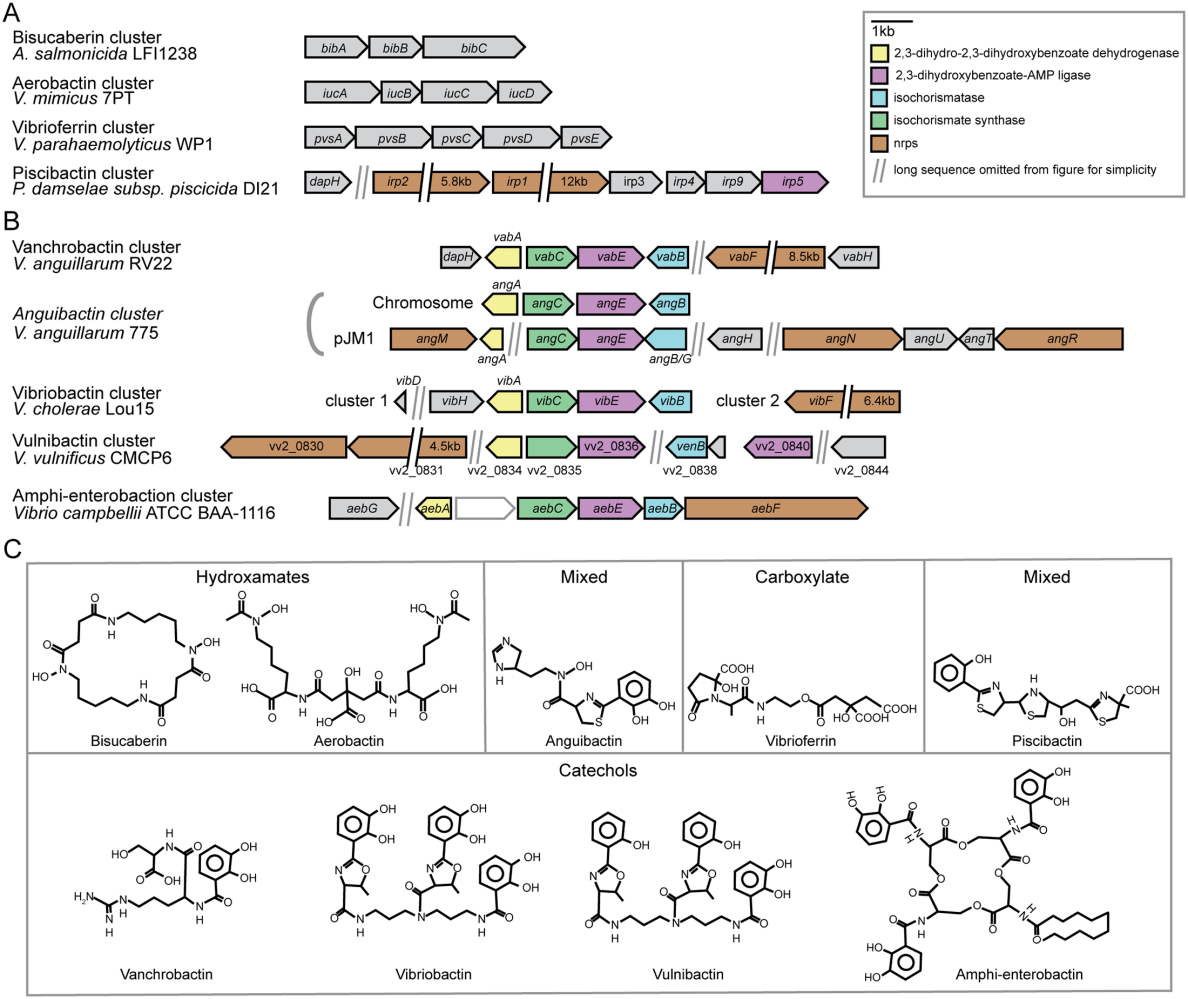
Organization of *Vibrionaceae* siderophore biosynthesis clusters and schematic structure of the corresponding siderophores. (A) *Vibrionaceae* hydroxamate and carboxylate and siderophore biosynthesis clusters. (B) *Vibrionaceae* catechol and mixed catechol/hydroxamate siderophore biosynthesis cluster. (C) Schematic 2D structure representation of *Vibrionaceae* siderophores with known biosynthesis gene clusters.

In this work, we set out to search both in the literature and the global sequence databases, to identify gene clusters for biosynthesis of siderophores in *Vibrionaceae*, and compile and visualize the result in a simple and comprehensible manner. See Materials and methods for details on search criteria. Fig 1 and Table 1 summarize our findings. Based on the literature we identified nine siderophore biosynthesis gene clusters responsible for producing aerobactin, bisucaberin, vibrioferrin, vibriobactin, vanchrobactin, piscibactin, anguibactin, amphi-enterobactin and vulnibactin. Fig 1 shows that genes belonging to the individual siderophore biosynthetic pathways are typically found clustered “bumper-to-bumper” on the chromosome, or as in *V*. *anguillarum*, on a plasmid. Pathways for hydroxamate or carboxylate type siderophores are encoded by 3–5 genes, all encoded on the same DNA strand, whereas catechol or mixed siderophores pathways are typically encoded by 6–11 genes, including one or more non-ribosomal peptide synthase (NRPS) gene(s), located on both strands and not necessarily in immediate proximity to each other. The synteny and general organization of the latter siderophore biosynthetic gene cluster types therefore appear more complex. Other siderophores are known to be produced by *Vibrionaceae* representatives. However, even though their structures are known, their biosynthesis gene clusters have not been conclusively identified in *Vibrionaceae*, and they have therefore been omitted from Fig 1. Examples are shown in S1 Fig, e.g., *V*. *fluvialis* and *Vibrio nigripulchritudo* produce the catechol siderophores fluvibactin and nigribactin, respectively [60,61]. Also, *Vibrio* isolates are known to produce trivanchrobactin (*V*. *campbellii* DS40M4; [62]), ochrobactins (*V*. sp. DS40M5; [63]) and desferroxamines (*V*. sp. BLI-41; [64]). In *V*. *campbellii* DS40M4 vanchrobactin and trivanchrobactin are produced from the same biosynthesis gene cluster, but in the distinguishing determinant is unknown[52].

Next, we used the known *Vibrionaceae* amino acids sequences (see Fig 1A and 1B) as queries in BLASTp searches to identify homologous siderophore gene clusters in all available *Vibrionaceae* genomes in the non-redundant protein sequences database. Threshold values were set to ≥80% coverage and ≥50% identity. Only complete siderophore biosynthesis clusters were kept (i.e., all genes needed for biosynthesis must be present). Our search identified 81 biosynthetic clusters in total, distributed among 45 species and 4 genera, and 16 unclassified *Vibrionaceae* strains (i.e., *Vibrio* sp.) (see S1 Table for details). The majority of species can potentially produce 1–3 of known *Vibrionaceae* siderophores, with zero being the minimum and four the maximum.

Bacteria must encode and express siderophore receptors on their surface in order to take up and utilize siderophore-Fe^3+^ complexes. It is therefore of equal importance to identify and map the existence of siderophore-associated receptors. In a similar approach as described above, we identified and used siderophore receptor sequences in BLASTp searches. (Table 2). The receptor searches identified 410 siderophore receptors in 89 classified *Vibrionaceae* species (and 49 unclassified *Vibrionaceae* strains), representing 5 genera (when using the same cut-off values as described above). The complete list of identified siderophore receptors is presented in S2 Table. We found homologs of known *Vibrionaceae* siderophore receptors in almost all *Vibrionaceae* species. Twenty-nine of the representatives in the split network do not encode homologs of known *Vibrionaceae* siderophore biosynthesis clusters or receptor. Of the 29, only 14 are fully sequenced, and the maximum number of different siderophore receptors found in a single genome was eight (i.e., in *V*. *alginolyticus*).

In summary, we searched the literature for known siderophore gene clusters from the *Vibrionaceae* family and identified nine types. The corresponding amino acids sequences were next used as queries in BLASTp to identify homologs. A total of 81 biosynthetic clusters distributed among 45 species and 16 unclassified *Vibrionaceae* strains were identified. Using a similar approach, we identified 409 siderophore receptor genes in 89 *Vibrionaceae* species and 49 unclassified *Vibrionaceae* strains.

### Distribution of siderophore biosynthesis clusters and siderophore receptors in the *Vibrionaceae* family

**Fig 2:**
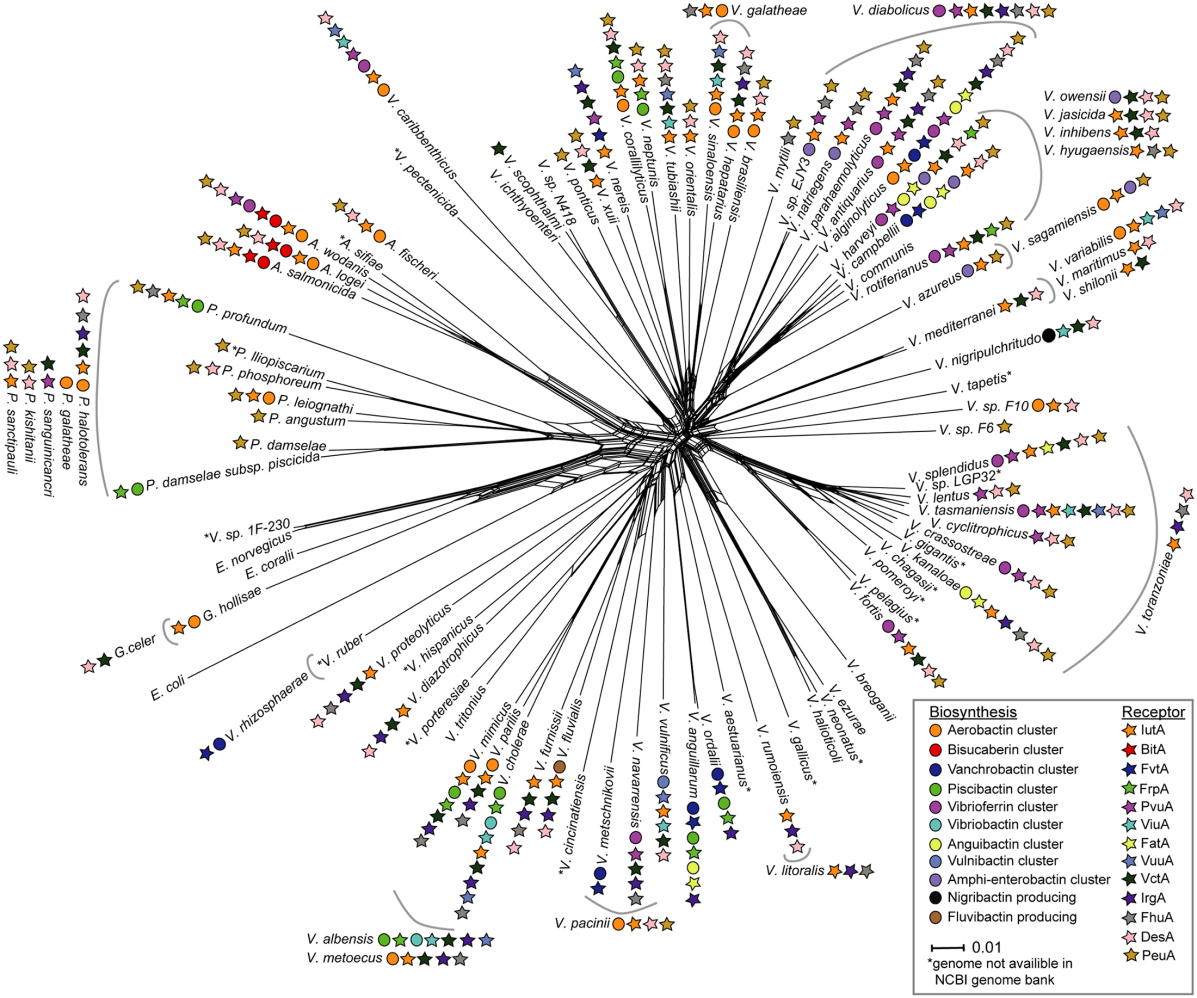
Distribution of homologs of known *Vibrionaceae* siderophore biosynthesis clusters and receptors mapped to a phylogeny. The phylogenetic split network is based on a dataset from Sawabe and co-workers [8], and consists of the genes *ftsZ*, *gap*, *gyrB*, *mreB*, *pyrH*, *recA*, *rpoA* and *topA*. The tree was constructed using SplitsTree4 to concatenate the individual gene alignments, and settings for network were uncorrected P and NeighborNet [56]. Branch lengths are to scale and species located outside grey arches were not included in the MLSA files and have been placed according to literature [71–86].

Fig 2 shows the distribution of siderophore biosynthetic systems and receptor genes on a phylogenetic network containing 86 representative species and unclassified strains from *Vibrionaceae*. Overall, the figure shows that the vast majority of species are associated with at least one type of siderophore system. We have, however not examined to what extent each of the siderophore systems are present in each species. In other words, individual isolates may or may not contain siderophore systems associated with that species, as indicated on the splits network. Moreover, some siderophore systems are restricted to a very narrow phylogenetic lineage, whereas others have a wide but sporadic presence. For example, the aerobactin, vanchrobactin and piscibactin biosynthesis clusters are scattered across multiple phylogenetic lineages, and anguibactin are found in *V*. *anguillarum* as well as in the *Splendius* and *Harveyi* clades. Similarly, vibrioferrin is found in *A*. *wodanis* and *Vibrio navarrensis*, and inside the *Harveyi* and *Splendidus* clades. A scattered distribution can potentially be explained (at least in part) by spread of siderophore clusters via plasmids. For example, serotype O1 strains of *V*. *anguillarum* 775 carries both chromosomal (Chr I) and plasmid-born (pJM1) genes for anguibactin biosynthesis, most of them on the plasmid (i.e., angB, angD, angCE, angN, angR, angM, angH, angT, angU) [40,65,66]. The plasmid-carried genes have been hypothesized to spread e.g., into *Vibrio harveyi*, or vice versa (see [67]). Similarly, a piscibactin biosynthesis cluster is located on a conjugative plasmid (pPHDP70) of a highly virulent *P*. *damselae* subsp. *piscicida* DI21 [50]. In experiments, Osario et al. showed that pPHDP70 can be conjugally transferred into multiple Gammaproteobacteria, including *E*. *coli*, *Aeromonas salmonicida*, and *V*. *anguillarum*. Moreover, the authors showed that a *V*. *alginolyticus* strain acquired the ability to both synthesize and utilize piscibactin after receiving pPHDP70 by conjugation.

To clarify if other *Vibrionaceae* representatives carry siderophore-encoding plasmids, we compiled all available plasmid sequences from the EBI Genomes Plasmid database (https://www.ebi.ac.uk/genomes/plasmid.html). These sequences were (i) submitted to antiSMASH ver. 4.0.2, and (ii) used as BLAST database in a tBLASTn search against all sequences from Table 1 as queries. The pPHDP70 plasmid sequence (described above) was missing from the EBI database and was manually added to the BLAST database file. Both methods identified the two plasmid-encoded systems in *V*. *anguillarum* and *P*. *damselae* subsp. *piscicida* as described above, but failed to find previously unrecognized plasmid-encoded siderophore gene clusters in *Vibrionaceae*.

To summarize, based on the current wide, but sporadic distribution of e.g., anguibactin and piscibactin, in addition to several lines of experimental evidence, it is likely that plasmids have contributed to transfers of siderophore gene clusters into new species, and thus likely contributed to the emergence of new pathogens due to increased capability to acquire iron from their surroundings. Extra care should therefore be taken, when comparing plasmid-borne and chromosomal-encoded siderophore gene clusters since their evolutionary histories can be complicated.

In contrast to the wide, but sporadic distribution described above, bisucaberin is narrowly distributed into one lineage, i.e., in three species from the *Fischeri* clade. This finding suggests that bisucaberin was introduced into *Vibrionaceae* through horizontal gene transfer into the most recent common ancestor of *Allivibrio*. Similarly, amphi-enterobactin is restricted to the *Harveyi* clade, vulnibactin is restricted to *V*. *vulnificus*, and vibriobactin is only found in the closely related species *Vibrio albensis* and *V*. *cholerae*. Interestingly, no siderophore biosynthesis clusters were identified in the *Halioticoli* clade.

In addition to showing presence/absence of siderophore biosynthetic gene clusters, Fig 2 also displays how the respective siderophore receptors are distributed in *Vibrionaceae*. Some main findings are that (i) the presence of biosynthetic genes for individual siderophores is accompanied by the presence of the corresponding receptor, (ii) the number of different types of receptors typically exceeds (and in some cases by far) the number of biosynthetic cluster types, and (iii) similar to the biosynthetic clusters the receptors are widely distributed in *Vibrionaceae*. E.g., *iutA* (aerobactin receptor gene) and *desA* (deferroxamine B receptor gene) are found in nearly all clades. Also, the receptor genes *viuA* (for vibriobactin), *vuuA* (for vulnibactin), *pvuA* (for vibrioferrin), *vctA*, *irgA* and *peuA* (all three for enterobactin), and finally *fhuA* (for ferrichrome) are widely distributed. In contrast, other receptors are more narrowly distributed, e.g., the bisucaberin receptor gene *bitA*, which is restricted to the *Fischeri* clade, more specifically to the same three *Aliivibrio* species that contain corresponding bisucaberin biosynthesis clusters.

Interestingly, (iv) known pathogens are conspicuously rich in siderophore receptors. E.g., *V*. *cholerae*, *V*. *alginolyticus* and *V*. *parahaemolyticus* encode seven, eight and five different receptor types, respectively. It is tempting to speculate that this richness likely reflects the lifestyle of these bacteria, where iron acquisition would be critical, especially during the initial phases of infections. Also, having multiple siderophore receptors would make them efficient “cheaters”, i.e., they can use siderophores produced by other species rather than from themselves. The receptors IrgA, VctA, FhuA, PeuA and DesA are found in many “cheaters” throughout *Vibrionaceae*. Another explanation for the apparent richness in receptor types is that these species have been characterized in more detail than environmental isolates, but at least multiple known pathogens still encode a higher number of known siderophore receptor types. It should however be noted that there are also examples of the opposite, i.e., very important pathogens that are poor in siderophore systems. E.g., the genome of *P*. *damselae* subsp. *piscicida* strain DI21encodes only one known siderophore system (piscibactin) (see Fig 2; [68]). Regardless, the bacterium is known as the causative agent of photobacteriosis, a disease that causes high mortality rates in outbreaks in fish farms worldwide (see [69]). According to a tBLASTn search from this study, the *P*. *damselae* subsp. *piscicida* strain OT-51443 genome [70] does not contain homologs of the piscibactin gene cluster found in strain DI21, or any other know siderophore cluster (known from *Vibrionaceae*). The sister subspecies, *P*. *damselae* subsp. *damselae*, also causes disease in a broader range of marine animals, and contains no known siderophore systems. It is possible that the apparent lack of siderophore-based iron uptake systems is compensated for by other systems, e.g., heme and/or hemoglobin uptake systems.

### Evolution of siderophore systems

To evaluate the evolutionary history of siderophore systems (biosynthesis and receptors) in *Vibrionaceae*, and to better understand their present-day distribution, we concatenated the protein sequences from the most abundant types of biosynthetic clusters separately, and aligned the resulting sequences using ClustalW. Only species included in Fig 2 were investigated. Maximum likelihood (ML) trees were generated from PvsABCDE (vibrioferrin), Irp123459 (piscibactin) and IucABCD (aerobactin) datasets. Similarly, datasets and ML-tree were constructed for siderophore receptors. The rationale for treating receptor sequences separate from biosynthesis genes was that receptor genes are often located elsewhere in the genome, and are much more widely distributed than the biosynthesis genes. ML-trees of the concatenated biosynthesis proteins and receptors were juxtaposed a host phylogeny based on same dataset as that used in Fig 2. Similar tree topologies (congruence) were interpreted as same evolutionary trajectories (i.e., vertical evolution), whereas conflicting topologies would suggest horizontal gene transfer events.

**Fig 3:**
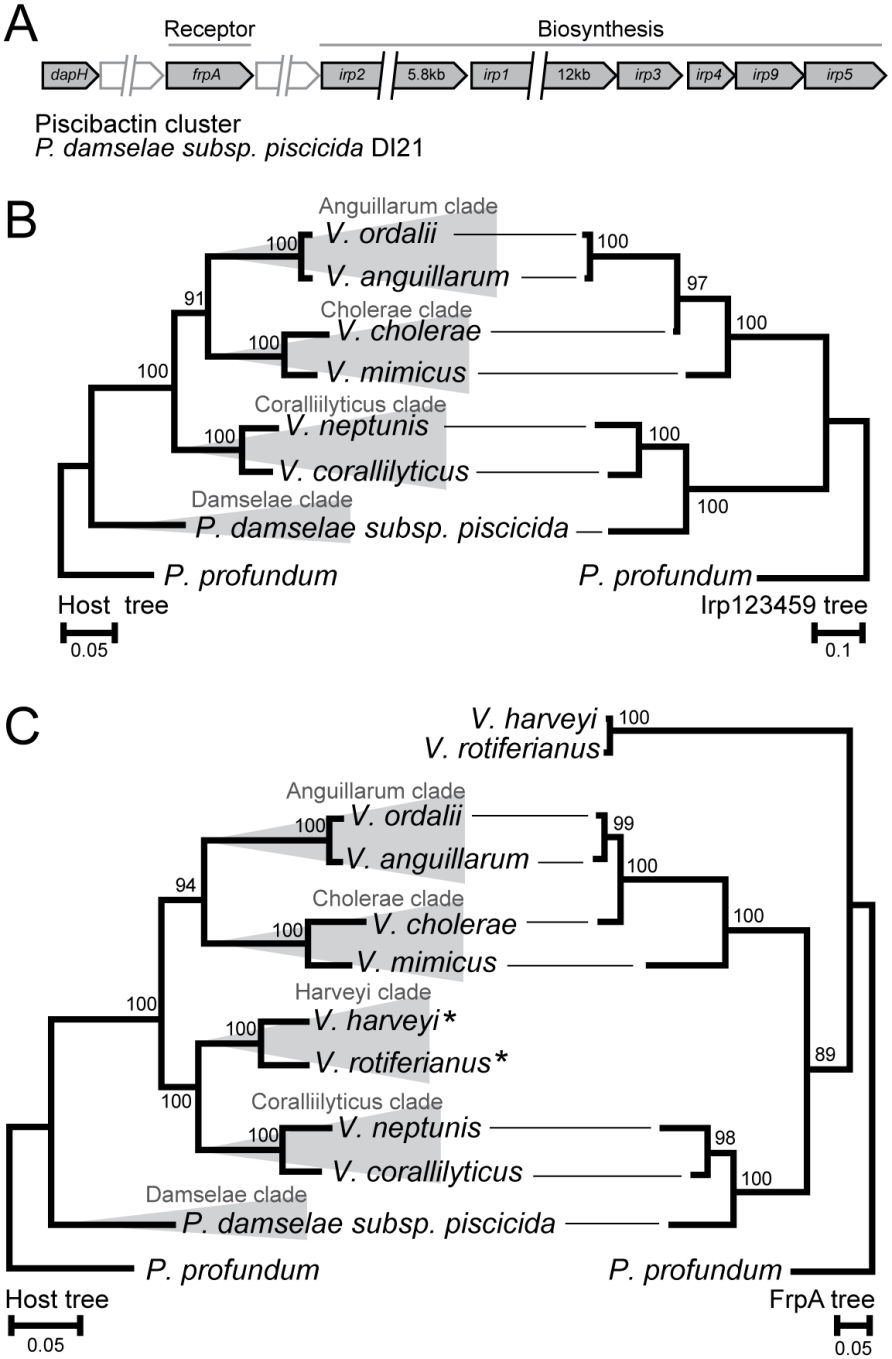
Phylogeny of the piscibactin biosynthesis cluster and receptor within the *Vibrionaceae* family. (A) The cluster organization of the biosynthesis cluster and the cognate receptor. (B) Host phylogeny on the left and piscibactin biosynthesis system (Irp123459) phylogeny on the right. (C) Host phylogeny on the left and piscibactin receptor (FrpA) phylogeny on the right. Asterisks denote species that do not encode the piscibactin biosynthesis system, i.e., the FrpA homolog is an exogenous siderophore receptor. Evolutionary analyses were conducted in MEGA6 [58]. The host trees were generated using the ML method and the TM model [59]. The siderophore biosynthesis cluster and receptor trees were generated using the ML method and the JTT model [87]. Bootstrap values are shown at the nodes (JTT model, 2000 replicates) [88]. Branch lengths are measured substitutions per site.

Fig 3 shows the genic organization and phylogeny of the piscibactin system. Nodes in the trees are highly supported by 95–100% bootstrap values. Although there are some discrepancies in the phylogenies, the overall tree topologies are very similar. Based on the criteria described above the data thus suggest that the piscibactin biosynthesis pathway was introduced early into *Vibrionaceae* and then stably inherited in a few lineages, and lost from the majority of lineages. Similarly, the overall topology for the proposed piscibactin receptor FrpA and the corresponding host tree are in good overall agreement, except for one clear case of misplacement, i.e., *V*. *harveyi* and *Vibrio rotiferianus* (*Harveyi* clade). Interestingly, these two species only contain the receptor, and not the biosynthesis system. This strongly suggests one horizontal gene transfer event of the FrpA receptor into the common ancestor of these two closely related species.

**Fig 4:**
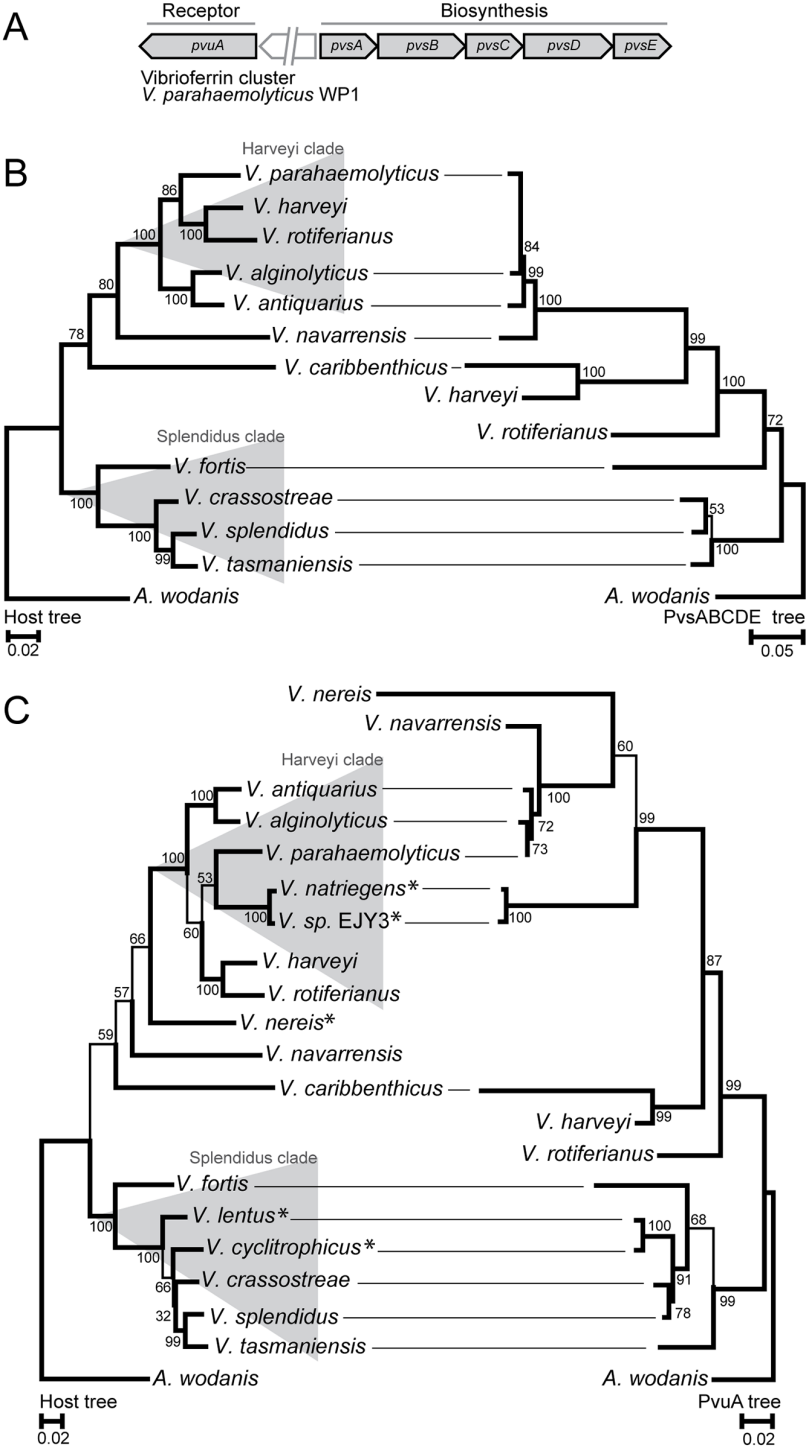
Phylogeny of the vibrioferrin biosynthesis cluster and receptor within the *Vibrionaceae* family. (A) The cluster organization of the biosynthesis cluster and the cognate receptor. (B) Host phylogeny on the left and vibrioferrin biosynthesis system (PvsABCDE) phylogeny on the right. (C) Host phylogeny on the left and vibrioferrin receptor (PuvA) phylogeny on the right. Asterisks denote species that do not encode the vibrioferrin biosynthesis system, i.e., the PuvA homolog is an exogenous siderophore receptor. Evolutionary analyses were conducted in MEGA6 [58]. The host trees were generated using the ML method and the TM model [59]. The siderophore biosynthesis cluster and receptor trees were generated using the ML method and the JTT model [87]. Bootstrap values are shown at the nodes (JTT model, 2000 replicates) [88]. Branch lengths are measured substitutions per site.

Fig 4 shows the genic organization and phylogeny of the vibrioferrin system. Intriguingly, the result is strikingly similar to that of the piscibactin system. The overall tree topologies for the biosynthesis system and the host phylogenies are very similar, except that *V*. *harveyi* and *V*. *rotiferianus* are clearly misplaced (strongly supported by high bootstrap values). The evolution of the associated receptor (PvuA) appears to be more influenced by horizontal gene transfer events. The PvuA and host trees are mostly congruent within the *Splendidus* clade, whereas the remaining branches have multiple clear, highly supported, misplacements in the PvuA tree (compared to the host tree). Therefore, the evolution of the biosynthesis and receptor genes is, in part, different with partly vertical and horizontal gene transfers.

**Fig 5:**
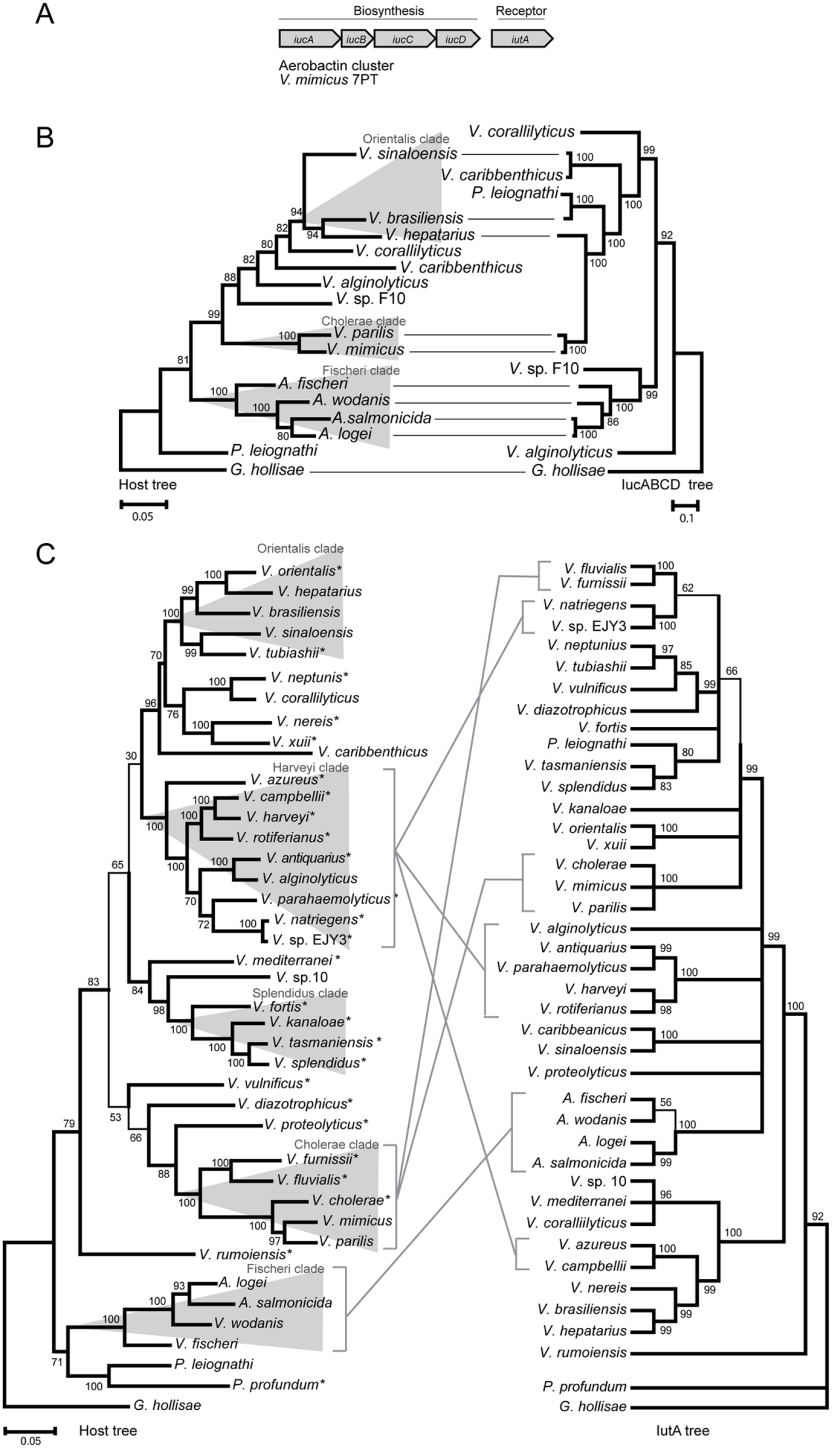
Phylogeny of the aerobactin biosynthesis cluster and receptor within the *Vibrionaceae* family. (A) The cluster organization of the biosynthesis cluster and the cognate receptor. (B) Host phylogeny on the left and aerobactin system (IucABCD) phylogeny on the right. (C) Host phylogeny on the left and aerobactin receptor (IutA) phylogeny on the right. Asterisks denote species that do not encode the aerobactin biosynthesis system, i.e., the IutA homolog is an exogenous siderophore receptor. Evolutionary analyses were conducted in MEGA6 [58]. The host trees were generated using the ML method and the TM model [59]. The siderophore biosynthesis cluster and receptor trees were generated using the ML method and the JTT model [87]. Bootstrap values are shown at the nodes (JTT model, 2000 replicates) [88]. Branch lengths are measured substitutions per site.

Fig 5 shows the genic organization and phylogeny of the aerobactin system. Nodes in the host and IucABCD trees are in general strongly supported by high bootstrap values (Fig 5B). In line with result from piscibactin and vibrioferrin phylogenies, comparison of the host and aerobactin tree topologies show both congruencies and conflicts, which suggests a mix of stable vertical inheritance, and cases of horizontal gene transfers. The evolution of its receptor (IutA) is however, much more complicated (Fig 5C). First, many nodes in the IutA tree are poorly supported. For clarity, the presented IutA tree is therefore a cladogram in which all nodes with less than 60% bootstrap support have been collapsed (60% majority-rule). Regardless, the host-IutA tree comparison reveal a high proportion of well supported conflicts, some of which are highlighted in the figure. Peculiarly, even representatives of closely related representatives from the *Harveyi* clade are found scattered at three different locations in the IutA tree, which suggest rampant spread of IutA within *Vibrionaceae*. Alternatively, the seemingly disordered IutA tree is a result of some artefact in our analysis. The IutA sequences were retrieved from protein databases using a conservative threshold setting (i.e., 50% identity/ 80% coverage), which suggest that the sequences are indeed homologs. Errors could potentially come from wrong naming of species in the databases, but even some errors in naming cannot explain the huge number of “misplacements” in the IutA tree. We therefore conclude that the IutA receptor has a complicated evolutionary history in *Vibrionaceae*, and has likely been introduced into the family several times, and/or been subjected to multiple horizontal gene transfers between *Vibrionaceae* representatives.

The narrow distribution of the bisucaberin cluster (in the *Fischeri* clade) suggests a different evolutionary history, i.e., a recent insertion event into a common ancestor of *A*. *salmonicida*, *A*. *wodanis* and *Aliivibrio logei*. Until recently, the bisucaberin biosynthesis genes (*bibABC*) were found exclusively in *A*. *salmonicida* (within *Vibrionaceae*) [48,89]. Here, the system is located on a genomic location (island) flanked by transposable elements. Our current BLASTp searches show that similar clusters are also found in *A*. *logei* and *A*. *wodanis*, together with the corresponding receptor gene *bitA*. So, where does this system originate from? We have in vain tried to identify the donor organism by running BLASTp and PSI-BLAST searches. The best database hits point to *Shewanella* as a possible source (BibA and BibB has 57% and 60% identity over 98% and 97% coverage, respectively, to *S*. *algae*. BibC 60% identity over 74% coverage to *Shewanella baltica* and *Shewanella putrefaciens*), but this needs to be addressed again as more genomic data from environmental marine bacterial strains are added to the databases.

In summary, the present-day distribution of siderophore systems in *Vibrionaceae* appears to be, perhaps as can be expected, a result of a combination of events: both old and new gene acquisitions, extensive gene loss, and both vertical and horizontal gene transfers.

## Supporting information

S1 Table Complete lists of homology hits from BLASTp query of nine *Vibrionaceae* siderophore biosynthesis clusters. Accession numbers and coverage/ identity/ e-value scores are included.
(XLSX)

S2 Table Complete lists of homology hits from BLASTp query of thirteen *Vibrionaceae* siderophore receptor. Accession numbers and coverage/ identity/ e-value scores are included.
(XLSX)

S1 Fig The 2D structure information for siderophores from *Vibrionaceae* without a known biosynthesis cluster. Structures of fluvibactin, nigribactin, trivanchrobactin, ochrobactins A-C and desferrioxamin G.
(PDF)

S1 Dataset Fasta file with MLSA dataset (used for construction of host tree in Fig 3B). (FAS)

S2 Dataset Fasta file with aligned and concatenated piscibactin biosynthesis clusters dataset (used for construction of Irp123459 tree in Fig 3B). (FAS)

S3 Dataset Fasta file with MLSA dataset (used for construction of host tree in Fig 3C). (FAS)

S4 Dataset Fasta file with aligned dataset of piscibactin receptor (used for construction of FrpA tree in Fig 3C). (FAS)

S5 Dataset Fasta file with MLSA dataset (used for construction of host tree in Fig 4B). (FAS)

S6 Dataset Fasta file with aligned and concatenated vibrioferrin biosynthesis clusters dataset (used for construction of PvsABCDE tree in Fig 4B). (FAS)

S7 Dataset Fasta file with MLSA dataset (used for construction of host tree in Fig 4C). (FAS)

S8 Dataset Fasta file with aligned dataset of vibrioferrin receptor (used for construction of FrpA tree in Fig 4C). (FAS)

S9 Dataset Fasta file with MLSA dataset (used for construction of host tree in Fig 5B). (FAS)

S10 Dataset Fasta file with aligned and concatenated aerobactin biosynthesis clusters dataset (used for construction of IucABCD tree in Fig 5B). (FAS)

S11 Dataset Fasta file with MLSA dataset (used for construction of host tree in Fig 5C). (FAS)

S12 Dataset Fasta file with aligned dataset of aerobactin receptor (used for construction of FrpA tree in Fig 5C). (FAS)

## Abbreviations

A. sp: *Aliivibrio* species
aa: Amino acid
ABC transporter: ATP-binding cassette transporter
BLASTp: Protein BLAST
G. sp: *Grimontia* species
ML: Maximum Likelihood
MLSA: Multilocus sequence alignment
NRPS: Non-Ribosomal Peptide Synthase
nt: nucleotide
P. sp: *Photobacterium* species
V. sp: *Vibrio* species
