# Supplementary figures and images for: Distribution of siderophore gene systems on a Vibrionaceae phylogeny: Database searches, phylogenetic analyses and evolutionary perspectives
Source: PLoS One. 2018 Feb 14;13(2):e0191860. doi: 10.1371/journal.pone.0191860 (PMC5812596; doi:10.1371/journal.pone.0191860)

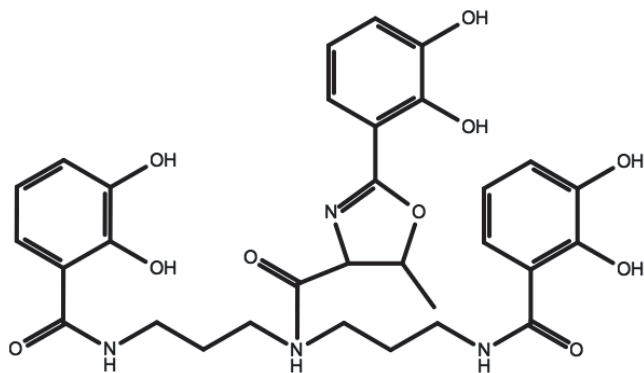

**Fluvibactin**

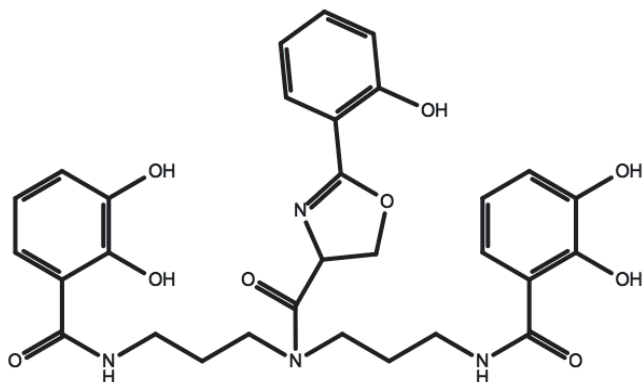

**Nigribactin**

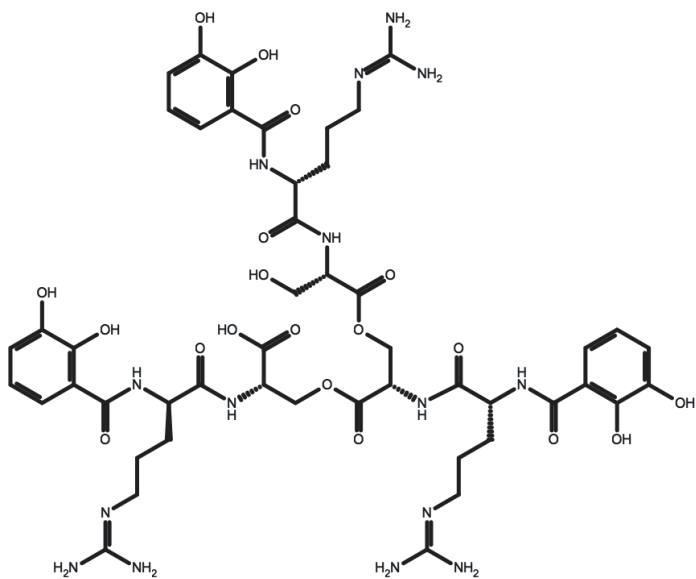

**Trivanchrobactin**

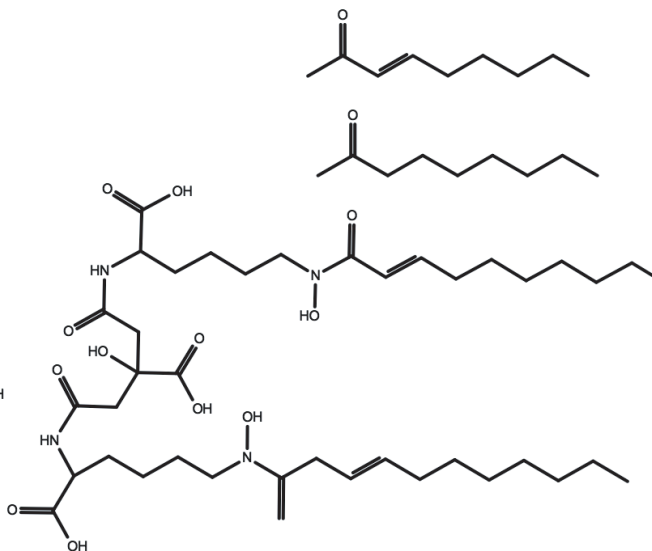

**Ochrobactins A-C**

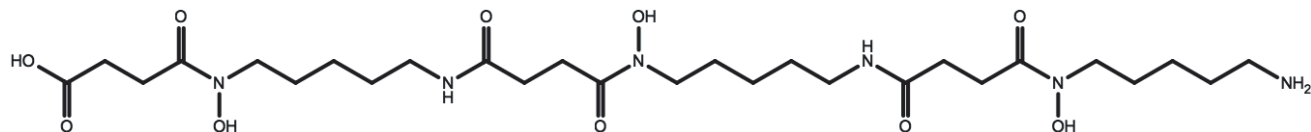

**Desferrioxamin G**

Supplement: S1 Fig — Structures of fluvibactin, nigribactin, trivanchrobactin, ochrobactins A-C and desferrioxamin G. (PDF) [file pone.0191860.s003.pdf]
